# The Role of *SOD1* and Oxidative Stress in Amyotrophic Lateral Sclerosis: An Overview

**DOI:** 10.3390/ijms27209084

**Published:** 2026-10-13

**Authors:** Carlos Alberto Gómez-Pérez, Miguel Ángel Ramírez-García, Petra Yescas-Gómez

**Affiliations:** 1 Departamento de Genética, Instituto Nacional de Neurología y Neurocirugía, Mexico City 14269, Mexico; 2 Posgrado en Ciencias Genómicas, Universidad Autónoma de la Ciudad de México, Mexico City 03104, Mexico

**Keywords:** SOD1, gene therapy, ROS

## Abstract

Amyotrophic lateral sclerosis (ALS) is a rapidly progressing neurodegenerative disorder that selectively affects motor neurons. The median survival time after diagnosis is 2 to 5 years. Oxidative stress (OS), driven mainly by reactive oxygen species (ROS), plays a central role in motor neuron (MN) degeneration. In this narrative review, we analyse the pathogenic role of SOD1 variants and their contribution to OS in ALS. SOD1 mutations alter the Cu/Zn superoxide dismutase enzyme, thereby promoting oxidative damage and triggering neuroinflammatory cascades via the MAPK, NF-κB and Nrf2/ARE signalling pathways. We also discuss emerging proteomic and machine-learning biomarkers, as well as the current therapeutic landscape. Riluzole and edaravone are approved treatments, while AMX0035 was approved in 2022 but withdrawn in 2024. Masitinib and EPI-589 remain investigational. In the realm of genetic therapies, the antisense oligonucleotide tofersen received accelerated FDA approval in 2023 for SOD1-ALS, primarily due to its ability to reduce plasma neurofilament light chain levels. However, the clinical benefit remains modest. RNA interference has reached human trials, while CRISPR/Cas9 remains in the preclinical stage. Both approaches have shown promising results in experimental models. Further research into ALS pathogenesis is needed to develop treatments that integrate antioxidant, anti-inflammatory, and gene-editing strategies.

## 1. Introduction

Aerobic organisms evolved to obtain energy more efficiently from the available oxygen (O_2_) in their environment. Due to the physicochemical properties of oxygen, these organisms are constantly exposed to oxidant agents, such as reactive oxygen species (ROS) and reactive nitrogen species (RNS), which play an essential role in cell development [1]. ROS are either radical or non-radical oxygen species that are formed by the partial reduction of O_2_ [2]. ROS contain O_2_ and its derivatives, which are generally very reactive and are partially reduced metabolites with oxidative capacities, although this may vary between species [3]. Examples of ROS include superoxide radical (O_2_^•−^), hydrogen peroxide (H_2_O_2_), and hydroxyl radical (^•^OH), among others [4]. Meanwhile, RNS are described as a group of nitrogen-bearing molecules with varying degrees of chemical reactivity, such as nitric oxide (NO^•^) [5].

ROS are primarily generated from the incomplete reduction of O_2_ during normal metabolism. These species can exist as either free radicals, which have unpaired electrons, or as non-radical species that are formed through radical–radical interactions. At physiological levels, ROS act as essential second messengers in cellular signalling pathways, regulating processes such as apoptosis, gene expression and ion transport. ROS can even promote health through a process known as mitohormesis. However, dysregulated antioxidant pathways have been associated with various diseases, and the high reactivity of ROS can damage macromolecules, including DNA, proteins, and lipids. ROS have also emerged as key regulators of cell death, determining whether cells undergo apoptosis, ferroptosis, or necroptosis and facilitating communication between these pathways [6,7,8]. Beneficial effects include roles in cellular responses to hypoxia, defence against infectious agents and various cellular processes such as differentiation, proliferation and immune responses [9]. However, excessive ROS accumulation can disrupt the redox balance, causing significant damage to cell structures, including lipids, membranes, proteins, and nucleic acids, which can lead to programmed cell death and result in oxidative stress (OS) [8]. To counteract these harmful effects, cells rely on antioxidant defence systems that preserve the redox balance and ensure cell survival [4]. Some authors suggest that OS is related to the development of pathologies such as Huntington’s disease, Alzheimer’s disease, Parkinson’s disease and amyotrophic lateral sclerosis (ALS), where an exacerbated oxidative tissue response is recognized [10,11].

Antioxidants can be classified in several ways. One common method is to divide them into primary and secondary antioxidants, depending on how they work. However, the human antioxidant system is more commonly divided into two categories: enzymatic and non-enzymatic. Additionally, synthetic antioxidants can be considered a separate group [12]. Endogenous enzymatic antioxidants are the body’s first line of defence against OS and nitrosative stress. Key enzymes such as superoxide dismutases (SODs), catalase (CAT), glutathione peroxidase (GPx), among others, catalyse reactions that detoxify ROS and RNS, thereby preserving redox balance and preventing macromolecular damage [13]. These enzymatic defences are particularly abundant in metabolically active organs such as the liver, brain, and kidneys, reflecting their critical role in maintaining tissue homeostasis under OS conditions [12,14].

On the other hand, non-enzymatic antioxidants act as free radical scavengers, metal chelators, and redox modulators. Molecules such as glutathione, uric acid, bilirubin, and coenzyme Q10 either directly neutralise ROS/RNS or regenerate oxidized enzymatic antioxidants, thereby reinforcing cellular antioxidant capacity. Their levels are tightly regulated through biosynthesis, recycling, and dietary intake [14]. Finally, exogenous antioxidants derived from the diet, including vitamins, polyphenols, carotenoids, and trace minerals, play a complementary role, particularly when endogenous defences are insufficient, as occurs during ageing, metabolic stress, toxin exposure, or chronic inflammation [15]. Together, these systems operate synergistically to preserve redox homeostasis and minimize the detrimental effects of OS on cellular functions.

## 2. Enzymatic Antioxidant System

**Figure 1:**
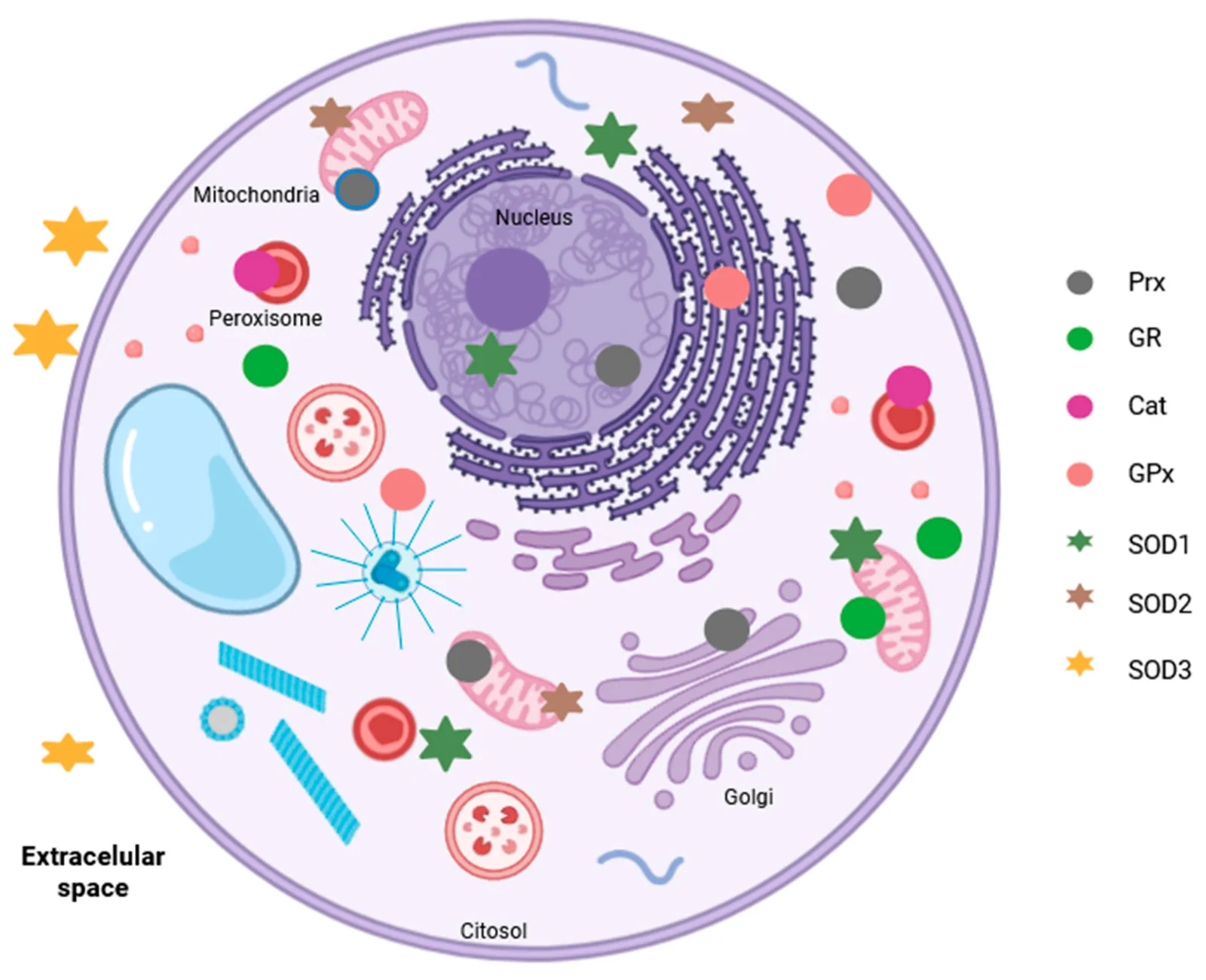
Subcellular distribution and compartmentalization of the main antioxidant enzyme systems. Schematic representation of the localization of defence mechanisms against OS within the cellular environment. *SOD1* (green star) is predominantly localized in the cytoplasm, the nucleus and the mitochondrial intermembrane space; SOD2 (brown star) is confined to the mitochondrial matrix, whereas SOD3 (orange star) acts mainly in the extracellular space. Also illustrated are the enzymes responsible for the clearance of H_2_O_2_ and the maintenance of the cellular redox state, including CAT (pink circle) in the cytoplasm and peroxisomes, GPx (light red circle), GR (green circle) and PRX (grey circle), distributed amongst the cytosol, mitochondria, endoplasmic reticulum and nucleus.

This system is composed of proteins with enzymatic activity that constitute the first line of defence against ROS and RNS (Figure 1), regulating cellular redox homeostasis:

- Superoxide dismutases (SODs): These are a group of metalloenzymes found in most aerobic organisms that serve as the first line of defence against free radicals. They catalyse the dismutation of the O_2_•^−^ into O_2_ and H_2_O_2_. SODs are classified according to their metal cofactors: Cu/Zn-SODs are found in the cytoplasm (SOD1) and extracellular space (SOD3). Mn-SOD (SOD2) is mainly located in mitochondria. The presence of specific SOD isoforms in different cellular compartments suggests tight spatial control over ROS homeostasis. Furthermore, evidence suggests that these enzymes participate in redox signalling networks between cellular compartments. Alterations in SOD1 enzymatic activity (principally mutations) can generate a localized H_2_O_2_ gradient, which can subsequently activate redox-sensitive pathways [16,17].
- Catalase (CAT): A haemoprotein that is primarily found in peroxisomes, where it catalyses the rapid decomposition of H_2_O_2_ into water and oxygen. This prevents the accumulation of H_2_O_2_, which could otherwise undergo Fenton reactions to produce highly reactive hydroxyl (•OH) radicals that are particularly damaging to lipids and DNA [16,18]. High CAT activity is observed in hepatic tissues, highlighting its importance in detoxification processes and hepatocellular protection [15].
- Glutathione peroxidases (GPx): GPxs are selenium-dependent enzymes primarily found in the cytoplasm and mitochondria. In the presence of reduced glutathione (GSH), they convert peroxides, such as H_2_O_2_ and organic hydroperoxides, into water and their respective alcohols. This limits lipid peroxidation [16,19]. GPx1 is widely expressed and plays a critical role in the antioxidant defences of the cardiovascular and nervous systems, while GPx4 specifically protects membrane lipids from peroxidation and is essential for embryonic development and the suppression of ferroptosis [20].
- Thioredoxin (Trx): Trx is a thiol-disulfide oxidoreductase that provides electrons to proteins such as ribonucleotide reductase and different peroxidases. It contributes to the elimination of ROS and RNS and participates in the regulation of redox signalling and apoptosis [17].
- Peroxiredoxins (PRXs): These form a large family of cysteine-dependent peroxidases. They possess a cysteine residue sensitive to oxidation by H_2_O_2_, and can efficiently reduce organic peroxides and peroxynitrites. In addition to their antioxidant function, PRXs act as redox sensors and modulators of intracellular signalling [17].

## 3. SOD1

The *SOD1* gene is located on chromosome 21q22.11 and spans 16.3 kb, comprising five exons. It encodes the canonical transcript NCBI RefSeq NM_000454.5 (corresponding to LRG_652t1) [21], which produces a mature cytoplasmic protein consisting of 154 amino acids. Although SOD1 is mainly a cytosolic protein, it has also been identified in the nucleus, lysosomes, peroxisomes, and the mitochondrial intermembrane space (Figure 1). This suggests that it may have additional functions in these compartments [22].

Structurally, mature SOD1 is a 32-kDa homodimer. Each 16-kDa monomer adopts an eight-stranded antiparallel β-barrel configuration with a Greek-key fold topology. Seven peripheral loops frame it, creating the active-site electrostatic channel and metal-binding pockets [23]. It also has a metal cofactor binding site and a global network of hydrogen bonds that help to maintain its stability [24,25]. The structural stability of SOD1 depends on the coordination of Zn^2+^ and Cu^2+^ ions, as well as the formation of a disulfide bond Cys57-Cys146 [26]. Zn^2+^ coordination stabilises the catalytic loop subdomains (loops IV and VII), while Cu^2+^ insertion, driven by the human copper chaperone for SOD1 (hCCS), promotes the oxidation of the Cys57-Cys146 disulfide bond. Furthermore, hCCS-dependent disulfide bond formation requires molecular oxygen, and hypoxic conditions hinder this process, thereby increasing structural disorder. Cleavage of this intramolecular disulfide bond and loss of metal cofactors result in a metal-free, denatured monomer, which constitutes the entry point to aggregation and filament formation in pathological states. Notably, Cys146 is particularly susceptible to overoxidation, becoming oxidized to sulfonic acid in contexts such as ALS, Alzheimer’s disease and Parkinson’s disease. This highlights its vulnerability and pathological significance [27].

SOD1 is a highly conserved enzyme, with 61.4% of its amino acids fully conserved, including all the residues involved in metal binding (His46, His48, His63, His71, His80, Asp83 and His120). This highlights its evolutionary stability [28]. However, recent quantitative analyses suggest that SOD1 protein levels in the human central nervous system (CNS) are closer to 0.16% of the total protein content, with variable distribution across peripheral tissues such as the liver, kidneys, red blood cells, and skeletal muscle [29]. In silico analyses have identified 233 mutations distributed throughout the SOD1 protein structure, including 31 in the metal-binding loop and 30 in the electrostatic loop. These mutations have the potential to impact protein folding and function [30].

### 3.1. SOD1 and OS in ALS Pathogenesis

ALS (OMIM: 105400) is an aggressive, adult-onset neurodegenerative disorder characterized by significant clinical, pathological, and genetic heterogeneity. It is primarily defined by the degeneration of motor neurons (MNs) in the cerebral cortex, brainstem and spinal cord, which results in progressive muscle atrophy and weakness. This can lead to total or partial paralysis and death within two to five years of symptom onset [31]. ALS has an estimated incidence of between 1.59 and 1.65 per 100,000 people per year, with a prevalence of between 4.42 and 5.05 per 100,000 people. The disease shows a clear age-dependent increase and is more prevalent among men [31,32]. The highest rates are observed in Europe and North America, while lower figures are reported in Asia and South America. Recent projections suggest that the prevalence of ALS will continue to rise over the coming decades. In Italy, for instance, it could reach 15.7 per 100,000 people by 2040. Meanwhile, in the United States, a rise of over 10% is anticipated by 2030, particularly among individuals aged 66 and over [33,34].

ALS mainly occurs sporadically (sALS). Only 5–10% of cases are familial (fALS) and primarily associated with the presence of genetic variants in multiple genes, such as *C9orf72*, *SOD1*, *TARDBP*, *FUS*, *VCP*, *ANG* and *PFN1*, amongst many others, with SOD1 being one of the most common. Both sALS and fALS exhibit a similar clinical course and neuropathological characteristics [35]. One common neuropathological mechanism is overwhelming evidence of OS, which has been suggested to be responsible for the degeneration and subsequent loss of MNs [36]. Other shared mechanisms include glutamate-induced cytotoxicity, dysregulation of neurotrophic factors, disruption of axonal transport, protein misfolding and aggregation, inflammation and mitochondrial dysfunction [37,38,39,40,41,42,43,44]. Studies have suggested that mild OS protects cells and tissues against infections and injuries. However, increased OS can lead to chronic inflammation. The main causes of this inflammation are the activation of glial cells in the CNS and the infiltration of peripheral immune cells. This inflammation contributes to the onset, progression and pathogenesis of ALS [36]. Nevertheless, caution is warranted when extrapolating findings from fALS models, particularly those involving *SOD1* gene variants (*mutSOD1*), to sALS. This is because the latter frequently involves complex gene–environment interactions that cannot be fully replicated in familial forms.

Various studies have highlighted the role of OS in ALS, identifying elevated levels of OS markers in the serum, cerebrospinal fluid (CSF), spinal cord, and muscle tissue of patients [45,46,47]. One such marker is 8-hydroxy-2′-deoxyguanosine (8-OHdG), which is indicative of oxidative DNA damage. Higher concentrations of this marker have been found in sALS patients compared with controls, reflecting genomic instability [48,49]. 4-hydroxynonenal (4-HNE), a toxic lipid peroxidation product, accumulates in serum and CSF, correlating with disease severity [11,48]. The 3-nitrotyrosine (3-NT) is a marker of peroxynitrite-mediated protein nitration. This compound is formed when O_2_^•−^ reacts with NO to create an unstable intermediate, ONOO^−^. This intermediate nitrates tyrosine residues and contributes to neurodegeneration [50]. While these molecules are robust indicators of oxidative damage at the molecular level, their potential as diagnostic or prognostic biomarkers is limited due to inconsistent correlations with the ALS Functional Rating Scale Revised (ALSFRS-R), and with disease duration and survival in small cohorts [49]. Therefore, they should mainly be interpreted as mechanistic readouts of OS rather than as validated clinical biomarkers for patient stratification.

Transgenic rodent and cell culture models expressing ALS-linked *mutSOD1* have been pivotal in elucidating the molecular pathology of OS in MN degeneration. Early mechanistic hypotheses postulated that toxicity stemmed from altered catalytic dismutation, either through superoxide accumulation due to loss of activity or dominant-negative inhibition of wild-type SOD1, or through excessive H_2_O_2_ production via catalytic hyperactivation [51].

**Figure 2:**
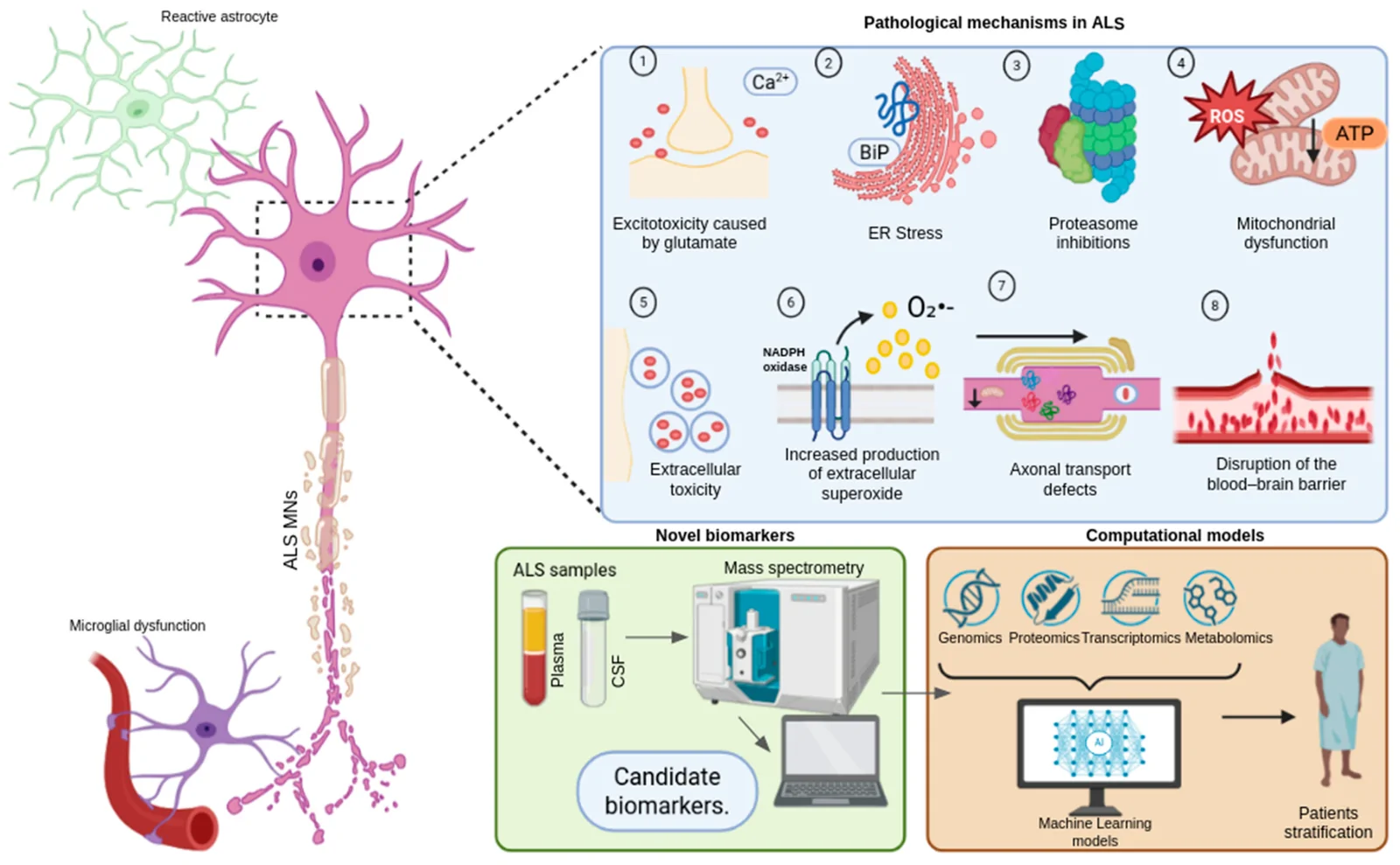
Pathological mechanisms and emerging biomarkers in ALS. MNs in ALS undergo degeneration driven by both cell-autonomous and non-cell-autonomous mechanisms. The left panel illustrates the major intracellular pathogenic pathways, including glutamate-mediated excitotoxicity, endoplasmic reticulum stress, proteasome inhibition, mitochondrial dysfunction, extracellular toxicity, increased production of extracellular superoxide, axonal transport defects and disruption of the blood–brain barrier. These interconnected processes converge to amplify oxidative stress, protein misfolding and neuronal vulnerability. The central panel shows how surrounding glial cells, such as reactive astrocytes and activated microglia, contribute to the disease by promoting neuroinflammation and exacerbating motor neuron injury through toxic crosstalk. The right panel shows emerging strategies for biomarker discovery that integrate proteomics, genomics, transcriptomics and metabolomics from ALS biospecimens (e.g., CSF and plasma). Computational models, including machine learning classifiers, enable patient stratification and the identification of candidate biomarkers. This bridges the gap between mechanistic insights and precision medicine approaches.

Identifying genetic variants in the *SOD1* gene was a milestone in our understanding of ALS pathogenesis, revealing that toxicity is caused not only by loss of function but also by the accumulation of abnormal properties. At least eight mechanisms (Figure 2) of toxicity have been described that illustrate the disease’s complex pathophysiology:

(1) Excitotoxicity caused by glutamate. In ALS, it arises from two mechanisms: the excessive release of glutamate at presynaptic terminals and impaired clearance of glutamate by astrocytes due to EAAT2 dysfunction. Evidence from transgenic SOD1 models suggests that even during the pre-symptomatic stage, the machinery responsible for releasing glutamate is hyperactive and there are fewer or incorrectly spliced astrocytic EAAT2 transporters. This results in persistently elevated levels of extracellular glutamate. This imbalance overstimulates NMDA and AMPA receptors, particularly calcium-permeable AMPARs lacking proper GluR2 editing. This promotes sustained Ca^2+^ influx, mitochondrial dysfunction, and oxidative stress. The convergence of these primary and secondary excitotoxic pathways amplifies motor neuron vulnerability and accelerates neurodegeneration [52,53].

(2) Endoplasmic reticulum (ER) stress. In ALS, *mutSOD1* tends to misfold and be deposited incorrectly within the ER. There, it interacts with resident chaperones, such as BiP, and proteins of the ER-associated degradation (ERAD) pathway. In particular, *mutSOD1* binds to Derlin-1, a key ERAD component. This blocks the retrotranslocation of misfolded proteins to the cytosol, thereby promoting their accumulation in the ER lumen. This persistent imbalance in protein homeostasis activates the unfolded protein response (UPR), including the PERK/eIF2α and IRE1/XBP1 pathways. These pathways attempt to restore homeostasis; however under chronic stress they drive pro-apoptotic cascades [54,55].

(3) Proteasome inhibition is another pathophysiological feature associated with *mutSOD1*. It has been suggested that this places a chronic burden on the ubiquitin–proteasome system, thereby impairing substrate degradation and reducing catalytic activity. This dysfunction favours the accumulation of ubiquitin-positive inclusions, a process that is further exacerbated by TDP-43 aggregates, which sequester proteasome components. Furthermore, recent evidence indicates that FMRP modulates TDP-43/TNKS/PI31 signalling, linking altered proteasome regulation to increased aggregation and neuronal vulnerability [56,57].

(4) Mitochondrial dysfunction is caused by the build-up of *mutSOD1* in the outer mitochondrial membrane. This disrupts the organelle’s morphology, its ability to buffer calcium and its ATP production. These alterations compromise mitochondrial dynamics, including the balance of fission and fusion, and impair respiratory chain activity, leading to the generation of excessive ROS. The resulting oxidative stress further destabilises mitochondrial membranes and exacerbates Ca^2+^ overload, creating a vicious cycle that accelerates motor neuron degeneration [58,59].

(5) Extracellular toxicity. The pathogenic effects of *mutSOD1* extend beyond the intracellular environment due to its abnormal secretion, which is often mediated by chromogranins A/B and non-canonical secretory pathways. Unlike wild-type SOD1, which acts as an antioxidant upon release, the secretion of *mutSOD1* is associated with cytoplasmic inclusions, microglial activation and the propagation of inflammatory cascades. Extracellular vesicles contribute to this process by transporting misfolded proteins and pro-inflammatory mediators, thereby amplifying neuroinflammation and accelerating motor neuron degeneration [52,60].

(6) Increased production of extracellular superoxide. *mutSOD1* contributes to OS by increasing the production of extracellular superoxide and maintaining NADPH oxidase in an active, Rac1-dependent state. This converts NADPH oxidase into a ROS generator and alters extracellular secretion. While wild-type SOD1 released via non-canonical secretory pathways can have neuroprotective antioxidant effects, the secretion of *mutSOD1* has been associated with cytoplasmic inclusions, microglial activation and motor neuron toxicity. This dual mechanism amplifies extracellular ROS generation and inflammatory signalling, thereby exacerbating neuronal vulnerability and disease progression [61].

(7) Axonal transport defects. In ALS, alterations to the cytoskeleton and vesicular trafficking can disrupt anterograde and retrograde transport. This leads to the accumulation of neurofilaments and the loss of synaptic vesicles in distal terminals. These defects compromise neuromuscular transmission and are considered to be among the earliest events in disease progression. Abnormalities in mitochondrial transport reduce the number of mitochondria in the axon and destabilise Ca^2+^ homeostasis, while defects in vesicular transport promote protein accumulation and Golgi fragmentation. Genetic variants in genes such as *SOD1*, *TARDBP*, *FUS* and *C9orf72* can further exacerbate transport failure, thereby reinforcing MN vulnerability [62,63].

Finally, (8) the disruption of the blood–brain barrier has been documented as a consequence of endothelial dysfunction in spinal capillaries. This results in a loss of tight junction proteins, such as ZO-1, occludin and claudin-5. This breakdown in barrier integrity is an early and progressive feature of the disease. It allows for vascular leakage and microhaemorrhages, as well as the entry of blood-derived proteins, iron, and inflammatory mediators into the CNS [64]. This exposure exacerbates neuroinflammation and OS, thereby increasing the vulnerability of MNs and accelerating degeneration.

In addition to these intrinsic mechanisms, *mutSOD1* toxicity involves non-cell-autonomous processes. Astrocytes and microglia play a pivotal role in this process. Recent studies have shown that astrocytes derived from patients with ALS disrupt proteostasis by reducing autophagy and promoting the accumulation of p62 and SOD1, thereby increasing neuronal vulnerability [65]. Similarly, the aberrant activation of microglia has been reported to enhance the release of ROS and pro-inflammatory cytokines, thereby exacerbating motor neuron degeneration [66,67]. In this context, communication between neurons and non-neuronal cells is recognized as a key pathogenic axis: bidirectional signalling between vulnerable MNs and activated glia generates a toxic microenvironment that perpetuates OS and inflammation, accelerating disease progression. Furthermore, the dysfunction of the ubiquitin–proteasome pathway and autophagy constitutes another fundamental pathogenic axis. Mutations in genes such as *OPTN*, *SQSTM1/p62*, *UBQLN2* and *VCP* disrupt the degradation of misfolded proteins and promote the formation of insoluble aggregates, which are a hallmark of ALS [58,68].

Taken together, these observations suggest that oxidative stress is not merely a marker of damage but a modulator of ROS-dependent signalling cascades. In this context, SOD1 mutations play a central role. Three main pathways stand out: MAPK, Nrf2/Keap1-ARE and NF-κB.

The activation of the MAPK pathway, particularly p38-MAPK, has been reported to be associated with motor neuron degeneration and microglial hyperactivation in SOD1^G93A^ transgenic mice. This pathway is activated via a negative feedback loop: inflammatory mediators induce MAPK activation, which enhances the release of further pro-inflammatory mediators, resulting in increased neuroinflammation and deficits in axonal transport [69]. Furthermore, the Nrf2/Keap1-ARE pathway, which is responsible for coordinating the cellular antioxidant response, has been found to be less active in MNs carrying the *mutSOD1*. This reduction in activation limits the ability to detoxify and defend against ROS, accelerating astrocytic activation and subsequent neuronal death. In SOD1^G93A^ transgenic mice, Nrf2 deletion accelerates the onset of symptoms and reduces survival, confirming its protective role [70,71,72]. Finally, in conditions of OS, the NF-κB pathway is abnormally activated in MNs, astrocytes and microglia. This leads to chronic inflammatory responses and apoptosis. Using SOD1^G93A^ mice, it has been demonstrated that suppressing NF-κB reduces neuronal loss and glial reactivity, improves motor function, and prolongs survival. This highlights the relevance of NF-κB as a therapeutic target [73,74,75].

### 3.2. Emerging Biomarkers in ALS

Beyond traditional fluid biomarkers such as neurofilaments and chitinase proteins, recent proteomics-based approaches have identified reproducible molecular signatures in ALS across CSF, plasma, and postmortem tissue. Large-scale studies using the Olink^®^ platform and mass spectrometry platforms have identified proteins involved in processes such as neuroinflammation (e.g., CHIT1 and CHI3L1), extracellular matrix remodeling (e.g., GPNMB and TNFRSF12A), and metabolic stress (e.g., FABP4). Many of these proteins have been found to be associated with survival and functional decline [76,77]. Consolidated in 2017 and 2018, respectively, resources such as the FindMNDBiomarker program have further reinforced the consistency of these protein changes across biospecimens, enabling the prioritization of high-confidence candidates for clinical translation [78].

These proteomic findings have paved the way for computational methodologies to play a central role in biomarker discovery. Machine learning methods applied to proteomic datasets have notably demonstrated high diagnostic accuracy (AUC > 0.9) and prognostic value. These methodologies have also identified protein panels that can predict ALSFRS-R decline and survival [79]. A more recent plasma proteomics study was conducted using the Olink Explore 3072 platform. The study enrolled 231 patients with ALS and 384 control participants, identifying 33 differentially abundant proteins. The most abundant protein was NEFL. These proteins were found to be enriched in pathways related to skeletal muscle, neurons, and energy metabolism. A random forest model incorporating 17 of these proteins and clinical covariates correctly classified patients and controls, achieving an AUC of 0.983 across a held-out test set and two external validation cohorts. The model also detected proteomic changes in individuals up to a decade before symptom onset [80]. However, these cohorts were predominantly of European ancestry, and the authors emphasize the need for validation in more diverse populations. A recent review discusses applying machine learning and deep learning to neuroproteomics. The review notes that none of the proposed panels have yet been used to inform diagnosis or treatment decisions because all reported performance metrics are derived from retrospective analyses [81]. Combining these strategies with multi-omics integration enables the stratification of patients and the identification of molecular subtypes, thereby bridging the gap between biomarker discovery and precision medicine. Furthermore, emerging metabolomic studies demonstrate how systemic signatures, such as altered amino acid and acylcarnitine profiles, can provide insight into pathophysiology and therapeutic responsiveness [82].

Recent computational approaches have extended beyond proteomic profiling to employ machine learning classifiers and mathematical modeling to improve the identification of biomarkers in ALS. Studies that combine diffusion tensor imaging with support vector machines have achieved a moderate level of diagnostic accuracy (~77%), demonstrating the potential of machine learning to detect microstructural changes in the corticospinal tract [83]. Transcriptomic pipelines integrating ensemble algorithms such as random forest and XGBoost have identified reproducible gene candidates (e.g., *DCN*, *CD44* and *POLR2B*) with >95% accuracy. This highlights their potential for stratification and therapeutic monitoring [84]. Complementary mathematical frameworks, such as principal component analysis and neural networks, have been used to analyze serum and skin biopsy data and distinguish oxidative and inflammatory signatures. These strategies support the characterization of minimally invasive biomarkers [85]. Although these strategies are still in the exploratory stage and require validation in larger cohorts, they demonstrate how computational tools can integrate high-dimensional datasets, refine biomarker panels, and converge with proteomics to advance precision medicine in ALS.

Together, these approaches demonstrate the transformative potential of proteomics and machine learning in the identification of ALS biomarkers. Ideally, they would combine diagnostic, prognostic and predictive dimensions within a multi-omics framework to capture disease heterogeneity and guide personalized interventions [86]. This integrative rationale is also crucial for current gene therapy advances, where biomarker-guided patient stratification is essential for optimizing the efficacy of antisense oligonucleotides, RNA interference and CRISPR-based strategies.

## 4. ALS Therapy

**Figure 3:**
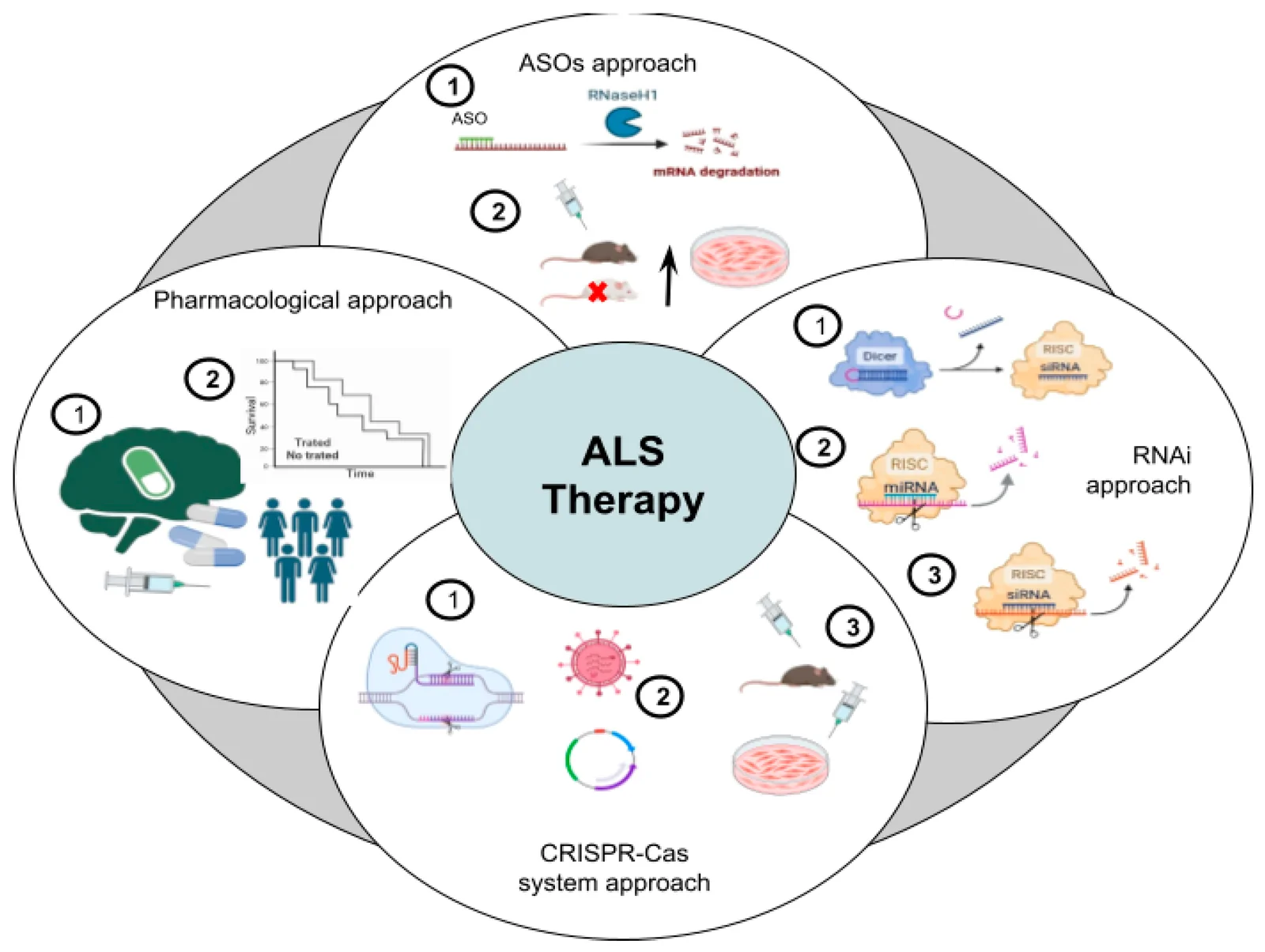
Therapeutic strategies for ALS focusing on mutant SOD1 pathomechanisms. Current therapeutic approaches and pharmacological and molecular gene-editing strategies are schematically represented: Pharmacological approach: (1) Administration of small molecules and systemic neuroprotective agents aimed at mitigating neuroinflammation, OS, and excitotoxicity; (2) Clinical translational evaluation highlighting survival extension curves in treated patients relative to non-treated controls. ASO approach: (1) Hybridization of single-stranded synthetic ASOs to target *SOD1* mRNA, recruiting RNase H1 to mediate targeted transcript degradation; (2) Functional validation demonstrating phenotype rescue, delayed disease onset and extended survival in transgenic ALS animal models and in vitro cell culture systems. RNAi approach: Gene silencing mediated by small non-coding RNAs: (1) Processing of precursor hairpins by Dicer and assembly into the RISC; (2–3) Post-transcriptional gene silencing via miRNAs or siRNA-loaded RISC complexes, promoting target mRNA cleavage and translational repression. CRISPR-Cas system approach: Direct genomic editing strategy: (1) Targeted double-strand breaks or base editing induced by gRNA-guided Cas nucleases targeting mutant *SOD1* loci; (2) Delivery strategies utilizing viral vectors (e.g., rAAV) or non-viral plasmid/RNP platforms; (3) Preclinical translation via direct intrathecal/systemic injection in ALS mouse models and validation in primary cell models.

Currently, there is no cure for ALS. However, various therapeutic strategies have been explored over recent decades with the aim of developing more effective interventions. Four main approaches stand out in this field (Figure 3): pharmacological therapies, therapies based on antisense oligonucleotides (ASOs), RNA interference (RNAi) and genome editing using the CRISPR-Cas system. These strategies seek to counteract various abnormal pathophysiological processes. This section describes the main studies focusing on treatments for individuals with *SOD1*-related ALS.

### 4.1. Pharmacological Therapies

Several pharmacological agents have been evaluated for the treatment of ALS. Riluzole (approved by the US FDA in 1995) and edaravone (approved in Japan and South Korea in 2015, in the US and Canada in 2017 and 2018, respectively) are approved therapies. In contrast, sodium phenylbutyrate–taurursodiol (PB-TURSO, AMX0035), approved in September 2022, was withdrawn from the market in 2024 following the failure of the Phase 3 PHOENIX trial to confirm its efficacy. Masitinib and EPI-589 remain investigational: masitinib is being evaluated in a confirmatory Phase 3 trial (AB23005) [87], while EPI-589 has only reached Phase 2. None of these agents have been shown to significantly halt or reverse disease progression or prolong life expectancy [88,89].

#### 4.1.1. Riluzole

An anti-glutamatergic drug approved by the FDA in 1995. As an ALS therapy, riluzole acts by inhibiting glutamatergic neurotransmission in the CNS through the inactivation of voltage-gated sodium channels and the activation of G-protein-dependent signal transduction [90]. More recent studies have shown that it may also be effective in inhibiting H_2_O_2_-induced OS in human spinal cord-derived astrocytes, which would contribute to its neuroprotective effect [91]. Riluzole is generally well tolerated, with the most common side effects including nausea, weakness and temporary increases in liver enzyme levels. However, its impact on survival and function appears to vary across populations. In the PRECISION-ALS study, a large multicentre European cohort, the use of riluzole was associated with a significant survival benefit, extending median survival by approximately seven months, particularly among patients with fast progression of the disease [92]. In contrast, a Chinese cohort study found no improvement in survival or ALSFRS-R decline, and subgroup analyses failed to identify responders [93]. Meanwhile, a US tertiary clinic study reported high initiation and adherence rates, with median use exceeding one year. Dose modifications were most often related to adverse effects, though social and economic factors also played a role [94]. Overall, these findings suggest that although riluzole remains the most widely prescribed therapy for ALS, its clinical benefits may depend on context. Survival advantages are more evident in Western cohorts, whereas limited efficacy is observed in Asian populations.

#### 4.1.2. Edaravone

Edaravone acts as a free radical scavenger, eliminating peroxyl and peroxynitrite radicals, and exhibits cytoprotective and neuroprotective properties in conditions involving OS. It protects neurons in the brain and spinal cord by eliminating ROS, which accelerate and exacerbate the progression of neuronal degeneration. This protects against the neurological damage that leads to the death of motor neurons [95]. Recent evidence, however, adds nuance to these findings. A systematic review and meta-analysis of over 2800 patients revealed that edaravone increased survival rates at 18, 24 and 30 months, though it had no significant impact on functional outcomes or adverse event rates [96]. In Germany, a large, multicentre, propensity-matched cohort study showed that long-term intravenous edaravone was feasible and generally well tolerated. However, it offered no disease-modifying benefit compared with standard therapy, regardless of whether patients met the original trial inclusion criteria [97]. Similarly, real-world data from the United States confirmed consistent safety and tolerability profiles, with discontinuation primarily driven by disease progression rather than adverse effects [98]. Overall, these findings suggest that edaravone is safe and widely tolerated across populations, but its clinical efficacy remains inconsistent. While some pooled analyses show survival benefits, functional outcomes are not consistently improved, and real-world studies in Western cohorts have failed to demonstrate robust disease-modifying effects. This highlights the need for further high-quality trials to clarify its role, particularly in relation to patient stratification and disease stage.

#### 4.1.3. Sodium Phenylbutyrate and Taurursodiol (PB-TURSO, AMX0035, Relyvrio)

This fixed-dose oral combination targets both ER stress and mitochondrial dysfunction, thereby reducing apoptosis and oxidative damage. Taurursodiol (TURSO) acts as a molecular chaperone, stabilising mitochondrial membranes and limiting ROS production. Meanwhile, sodium phenylbutyrate (PB) functions as a histone deacetylase inhibitor and chemical chaperone to support proteostasis. Preclinical studies have shown that this combination produces broader metabolic and transcriptomic changes than either compound alone. These changes affect mitochondrial function, the unfolded protein response, and RNA processing [99,100,101].

The CENTAUR Phase 2 trial demonstrated a slower decline in ALSFRS-R scores over a period of 24 weeks. Long-term analyses revealed survival benefits of between 6.5 and 18.8 months, and a reduced risk of death was observed among patients who received early, continuous treatment [102]. Further biomarker analyses indicated reductions in plasma YKL-40 and CRP, which correlated with slower disease progression [103]. However, the subsequent PHOENIX Phase 3 trial failed to confirm these benefits, resulting in the drug being withdrawn from the US and Canadian markets. Real-world studies have reported consistent safety, as well as frequent gastrointestinal adverse events and high discontinuation rates, which are often linked to cost and insurance barriers [104]. Overall, PB-TURSO remains mechanistically promising, but its efficacy is uncertain, with survival advantages largely confined to exploratory analyses rather than definitive trial evidence.

#### 4.1.4. Masitinib

Masitinib is a novel tyrosine kinase that acts on microglia and mast cells. It reduces neuroinflammation by inhibiting enzymes involved in cell proliferation and degranulation [105,106]. In a phase 2 clinical trial (NCT02588677), its efficacy in combination with riluzole was evaluated. The results showed improvements in the ALSFRS-R score, slower disease progression and better quality of life in patients receiving treatment [107]. These results suggest that inhibiting microglial activation could be an effective strategy for reducing neuronal damage in ALS.

#### 4.1.5. EPI-589

(R)-troloxamide quinone is a safe and well-tolerated inhibitor of oxidoreductase enzymes. In a Phase 2a clinical trial (NCT02460679), EPI-589 was shown to significantly reduce OS and neuroinflammation, while also slowing disease progression. These results suggest that synthetic compounds with antioxidant and anti-inflammatory properties could potentially modulate neuronal loss and glial activation in ALS. However, further research is needed to confirm their long-term efficacy [36,108].

Taken together, these pharmacological agents demonstrate a variety of approaches targeting the pathophysiological mechanisms of ALS. While some marginal benefits have been observed, such as limited prolongation of survival or reduced markers of oxidative damage, none have succeeded in significantly halting disease progression. These results emphasise the potential and limitations of pharmacological therapies for ALS, highlighting the need to explore complementary genetic and molecular strategies, such as antisense oligonucleotides, RNA interference, and genome editing via CRISPR-Cas9.

### 4.2. Antisense Oligonucleotides (ASOs)

ASOs are synthetic, single-stranded oligonucleotides that are typically 15–30 nucleotides long. They bind to target RNA via Watson–Crick base pairing, thereby modulating protein expression [109,110,111]. ASOs can target either mature mRNA or pre-mRNA and act through two main mechanisms. In the RNase H-dependent mechanism, which is used by gapmer ASOs (a central DNA segment flanked by modified nucleotides), RNase H cleaves the ASO–RNA hybrid. This process is catalytic, meaning a single ASO can contribute to the destruction of many transcripts. This mechanism was used in the development of ASOs for familial ALS. In the RNase H-independent mechanism, ASOs act by steric hindrance or by modulating splicing. For instance, they can mask regulatory elements in pre-mRNA, thereby inducing either exon skipping or inclusion [110,111].

Unmodified phosphodiester ASOs are rapidly degraded by nucleases [111], so successive chemical modifications were developed: phosphorothioate backbones (nuclease resistance, support for RNase H activity), 2′-sugar modifications such as 2′-O-methyl, 2′-O-methoxyethyl and locked nucleic acids (greater nuclease resistance and binding affinity), and conjugation to ligands to improve delivery [109,111]. Because ASOs generally do not cross the blood–brain barrier, they are given intrathecally in CNS disorders [111]. By contrast, siRNAs are double-stranded RNAs loaded into the RNA-induced silencing complex (RISC), where the antisense strand guides cleavage of the target mRNA. They tolerate mismatches more than gapmer ASOs, which increases off-target risk, and their double-stranded structure makes tissue delivery harder [110].

ASO-based therapy has shown potential in treating monogenic ALS, particularly in *SOD1* carriers. In April 2023, tofersen (BIIB067), an ASO that degrades *SOD1* mRNA, received accelerated FDA approval for *SOD1*-related ALS, primarily due to a reduction in plasma neurofilament light chain (NfL) levels [112]. A phase 1–2 trial involving five intrathecal doses of 20–100 mg over 12 weeks reduced CSF SOD1 by 36%, compared with a 3% reduction from placebo. However, there was no improvement in the ALSFRS-R score [113]. In the phase 3 VALOR trial, tofersen lowered CSF SOD1 and plasma NfL; however, after 28 weeks, ALSFRS-R scores were not significantly different to placebo. Participants who started treatment at an earlier stage of the disease experienced less decline than those who started during the open-label extension [114,115].

Preclinical studies have demonstrated the ability of ASOs targeting *SOD1* to modify the progression of the disease. Smith et al. [116] infused a 20-mer phosphorothioate/2′-O-methoxyethyl ASO rat *SOD1* into the ventricles at a dose of 100 µg per day for 28 days. This reduced *SOD1* mRNA levels in the brain and spinal cord to 25–40% of normal, and reduced SOD1 protein levels by 50% in the frontal cortex and the cervical and lumbar spinal cord. In SOD1^G93A^ rats, a human-targeting ASO did not slow early disease but extended survival (132 ± 7 vs. 122 ± 8 days), a 37% extension of the typical 7-day disease duration after onset. In rhesus monkeys, the same class of ASO (1 mg/day for 14 days) reached micromolar concentrations throughout the CNS, including lumbar motor neurons. In fibroblasts from a patient carrying the SOD1 A4V mutation, the ASO reduced human *SOD1* mRNA levels by around 80%.

Antisense sequences can also be delivered by viral vectors. Biferi et al. [117] used an AAVrh10 vector carrying an antisense sequence embedded in a modified U7 snRNA (AAV10-U7-hSOD), which induces skipping of *SOD1* exon 2. This leads to the transcript being degraded by nonsense-mediated decay. Combined intravenous and intracerebroventricular administration to SOD1^G93A^ mice at birth or at 50 days of age increased survival by 92% and 58%, respectively. The authors suggest that this approach could be applied to SOD1-linked familial ALS.

Overall, ASO-based approaches have demonstrated the ability to reduce *SOD1* expression directly in the CNS. However, the clinical benefits have been modest thus far, with changes in biomarkers (e.g., CSF SOD1 and NfL) proving to be more consistent than functional outcomes [113,115]. The need for repeated intrathecal administration has also led to the development of alternative strategies, such as RNA interference (RNAi), which could allow for less frequent dosing [112,115].

### 4.3. RNA Interference (RNAi)

RNAi is an endogenous gene-silencing mechanism in which small RNAs guide the RISC to complementary mRNAs. Small interfering RNAs (siRNAs) are fully complementary to their targets and lead to direct cleavage of the transcript, whereas microRNAs (miRNAs) are only partially complementary and mainly repress translation or destabilise the mRNA [118]. Unlike ASOs, siRNAs are double-stranded and require delivery systems to reach the CNS [112,115].

Xia et al. [119] identified two small hairpin RNAs (shRNAs) that inhibited over 95% of *SOD1* reporter expression, silencing both the wild-type and mutant (G93A and G85R) forms of *SOD1* in the process. As wild-type *SOD1* is essential for motor neuron health, the researchers proposed pairing the shRNA with an RNAi-resistant *SOD1* replacement gene. However, they noted that off-target effects, interferon responses and promoter choice needed to be tested in vivo. In SOD1 rodent models, increased survival was observed following silencing, but the extent to which this occurred depended on the vector, the route of administration and the age at which treatment was administered. For example, intramuscular injection of a retrogradely transported lentiviral shRNA at postnatal day 7 delayed disease onset and prolonged survival [120], whereas neonatal intravenous AAV9-shRNA increased median survival by approximately 39% [121]. Neonatal intracerebroventricular delivery of an AAV9-encoded microRNA increased median survival by around 50% and prevented limb paralysis [122]. Conversely, AAV6-mediated systemic or intramuscular shRNA delivery did not alter disease progression, despite efficient neuromuscular transduction [123,124]. Targeting individual cell populations was beneficial, but not sufficient; silencing in either motor neurons or glia improved neuromuscular function and survival [125], while suppression in the motor cortex of SOD1^G93A^ rats delayed onset and extended survival by around 20 days [126]. Delivery in adult animals proved more difficult. Intrathecal AAVrh10-miRNA increased survival by only around 11% in adult mice [127]. In contrast, systemic AAVrh10-mediated silencing delayed the onset of symptoms and death in adult mice, and intrathecal delivery was safe in non-human primates [128]. It was later shown that subpial AAV9-shRNA delivery achieves widespread silencing and blocks motor neuron degeneration in SOD1^G37R^ mice. This finding was validated in larger animal models [129]. More recently, double-stranded RNA interference targeting *SOD1* was shown to nearly double median survival in ALS mice (210 vs. 128 days) [130]. Together, these studies suggest that widespread transduction of motor neurons and glia is the main obstacle to effective RNAi [122].

Clinical translation has only recently begun. AMT-162 (APB-102), which is an AAV-delivered miRNA that targets *SOD1*, has been administered intrathecally to two patients and is currently being evaluated in a phase 1/2 trial [115,118]. Chen et al. [112] developed RAG-17, an siRNA conjugated to an accessory oligonucleotide to improve CNS delivery. In SOD1^G93A^ mice, RAG-17 increased survival even when treatment began well after symptom onset (by 46.3% and 75.8% at 200 µg and 400 µg, respectively), and produced sustained *SOD1* knockdown in non-human primates. In a phase 1 trial involving six patients with SOD1-ALS, intrathecal administration of RAG-17 reduced CSF *SOD1* by 56–69% and plasma NfL by 52–62%. Only mild to moderate adverse events were observed, with no serious adverse events reported. However, this trial was open-label and very small, and the authors acknowledge that larger, controlled studies are needed to establish efficacy.

RNAi-based approaches are also being explored for other ALS genes. AAV-delivered miRNA molecules have been developed for *C9orf72* [115,118], and modulation of miRNAs, such as the inhibition of miR-155, has extended survival in SOD1^G93A^ mice [131,132]. Overall, RNAi offers potent and potentially durable silencing, but challenges remain regarding delivery, off-target effects and long-term safety.

### 4.4. CRISPR-Cas Systems

Since the 1990s, various strategies have been developed to carry out specific gene editing in different cell types and organisms. These strategies aim to correct pathogenic mutations, inactivate target genes, or introduce new genetic traits [133,134,135]. The CRISPR (clustered regularly interspaced short palindromic repeats)-Cas (CRISPR-associated) nuclease system is the third most widely used genome-editing tool worldwide [136].

The CRISPR-Cas system is an adaptive immune system inherent to prokaryotic organisms, such as bacteria and archaea. It protects them against infections caused by phages, viruses and other exogenous elements [137,138]. The system comprises CRISPR repeats, spacers and Cas genes, which are transcribed into CRISPR RNA (crRNA), trans-activating CRISPR RNA (tracrRNA) and Cas nucleases, respectively. The system is classified into two classes (I and II) and six types (I–VI), which differ mainly in the number of effector proteins involved in cleavage. Class I comprises multiprotein complexes (types I, III and IV), whereas class II comprises a single effector protein (types II, V and VI) [139,140].

The CRISPR-Cas9 system is characterized by its high efficiency and speed in gene editing, enabling its application to a wide variety of organisms. In the medical field, it is mainly used to generate animal and cellular models of human diseases, including knockout, knock-in and targeted mutagenesis models [141,142].

In recent years, CRISPR-Cas9 has been employed as a therapeutic approach for neurodegenerative diseases, including ALS. In 2017, Gaj et al. [143] demonstrated that the system could reduce the expression of the *mutSOD1* protein using SaCas9, a Cas9 protein derived from *Staphylococcus aureus.* In NSC-34 cells, the sgRNA that most efficiently targeted exon 2 of the hSOD1^G93A^ transgene reduced expression of the mutant protein by ~92%. In SOD1^G93A^ neonatal mice treated with AAV9-SaCas9-hSOD1, a significant reduction in *SOD1* expression in the spinal cord was observed, alongside a delay in disease onset (33 days), increased survival (an additional 28–30 days), and improvements in motor function and muscle mass. These results confirmed the therapeutic benefit of CRISPR-Cas9-mediated gene editing in ALS models.

Meanwhile, Deng et al. [144] evaluated the long-term safety of gene editing using mouse models. They introduced an sgRNA targeting exon 2 of hSOD1 into mouse embryos and generated founder strains, which were then crossed with SOD1^G93A^ mice. The double-transgenic mice (G1H-Cas9 and G1L-Cas9) exhibited normal phenotypes and prolonged survival (approximately 32 and 8 months, respectively), in contrast to the controls, which developed ALS within 3–4 months. These results imply that the CRISPR-Cas9 technique could be developed into an effective therapeutic strategy for rapidly progressive degenerative diseases such as ALS, despite the associated risks.

Preclinical studies have demonstrated that gene editing using CRISPR-Cas9 can significantly reduce the expression of mutant proteins, delay clinical progression and improve function in ALS models. However, challenges remain regarding the efficiency of vector delivery, long-term safety and the potential for off-target effects. This highlights the need for further research before CRISPR-Cas9 can be widely used in clinical practice [145].

## 5. Limitations of Antioxidant Therapies and Interventions in ALS

Despite advances in pharmacological and molecular therapies for ALS, there is currently no strategy that has halted or reversed disease progression. Riluzole prolongs survival by a few months, while edaravone slows functional decline in a subset of patients, but does not demonstrate a survival benefit. AMX0035, a combination of sodium phenylbutyrate and taurursodiol, showed promise in an early trial, but the phase 3 trial failed to confirm these results, resulting in the withdrawal of the drug in 2024. Masitinib and EPI-589 remain investigational, with evidence limited to specific patient subgroups or early-phase studies.

Genetic therapies have progressed unevenly. ASOs have been approved for use in treating SOD1-ALS (tofersen), but the clinical benefit has so far been modest compared with the consistent changes in biomarkers such as CSF SOD1 and plasma NfL. RNAi has only reached first-in-human trials, and CRISPR-Cas9 remains in the preclinical stage. These approaches share significant limitations in terms of biodistribution, invasive administration, long-term safety, and off-target effects. Moreover, their efficacy depends on known mutations. SOD1 variants account for approximately 15–30% of familial cases and up to 2% of all ALS cases, which limits their applicability to sporadic cases.

Overall, it seems that no single strategy is sufficient, and that ALS treatment will require a combination of personalized pharmacological, antioxidant and molecular approaches.

## 6. Conclusions

ALS remains a devastating disease with a multifactorial aetiology involving processes such as OS, excitotoxicity, mitochondrial dysfunction, impaired proteostasis, and neuroinflammation. Despite advances in pharmacological therapies, the clinical impact has been limited. Riluzole and edaravone provide only modest benefits; AMX0035 was withdrawn in 2024; and masitinib and EPI-589 remain investigational. Molecular strategies have progressed more rapidly. The ASO tofersen has received accelerated approval for SOD1-ALS, although its clinical benefit has so far been modest compared with its impact on biomarkers such as CSF SOD1 and plasma NfL. RNAi has reached first-in-human trials, and CRISPR-Cas9 has shown promising results in preclinical models, particularly in *mutSOD1* models. In parallel, recent proteomic and machine-learning approaches show potential to expand the biomarker landscape and refine patient stratification, thereby supporting precision medicine. However, these approaches still require validation in diverse populations. Caution is also needed when extrapolating findings from fALS models, particularly *mutSOD1* models, to sALS, due to the heterogeneity and complex gene–environment interactions underlying most cases. Overall, the evidence suggests that an effective ALS treatment will require strategies that target multiple pathogenic mechanisms. Future research should therefore focus on combined and personalised therapies that modulate basic cellular processes and specific genetic alterations. The ultimate goal is to alter the natural progression of the disease and provide patients with new treatment options.

## Data Availability

No new data were created or analyzed in this study. Data sharing is not applicable to this article.
